# Supplementary figures and images for: Conservation and divergence of mitochondrial apoptosis pathway in the Pacific oyster, Crassostrea gigas
Source: Cell Death Dis. 2017 Jul 6;8(7):e2915–. doi: 10.1038/cddis.2017.307 (PMC5550854; doi:10.1038/cddis.2017.307)

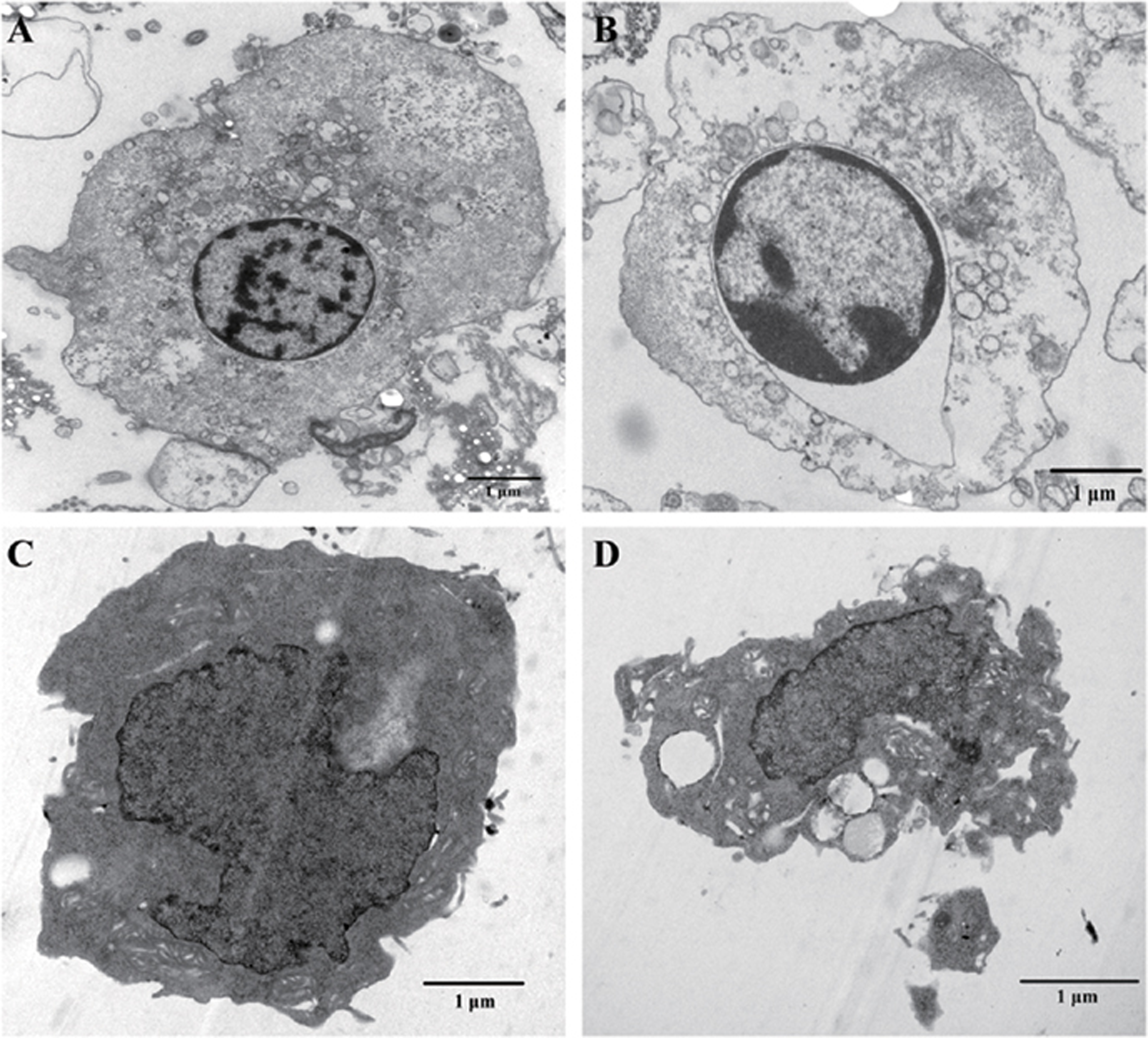

Supplement: Supplementary Figure 1 [file cddis2017307x1.tif]

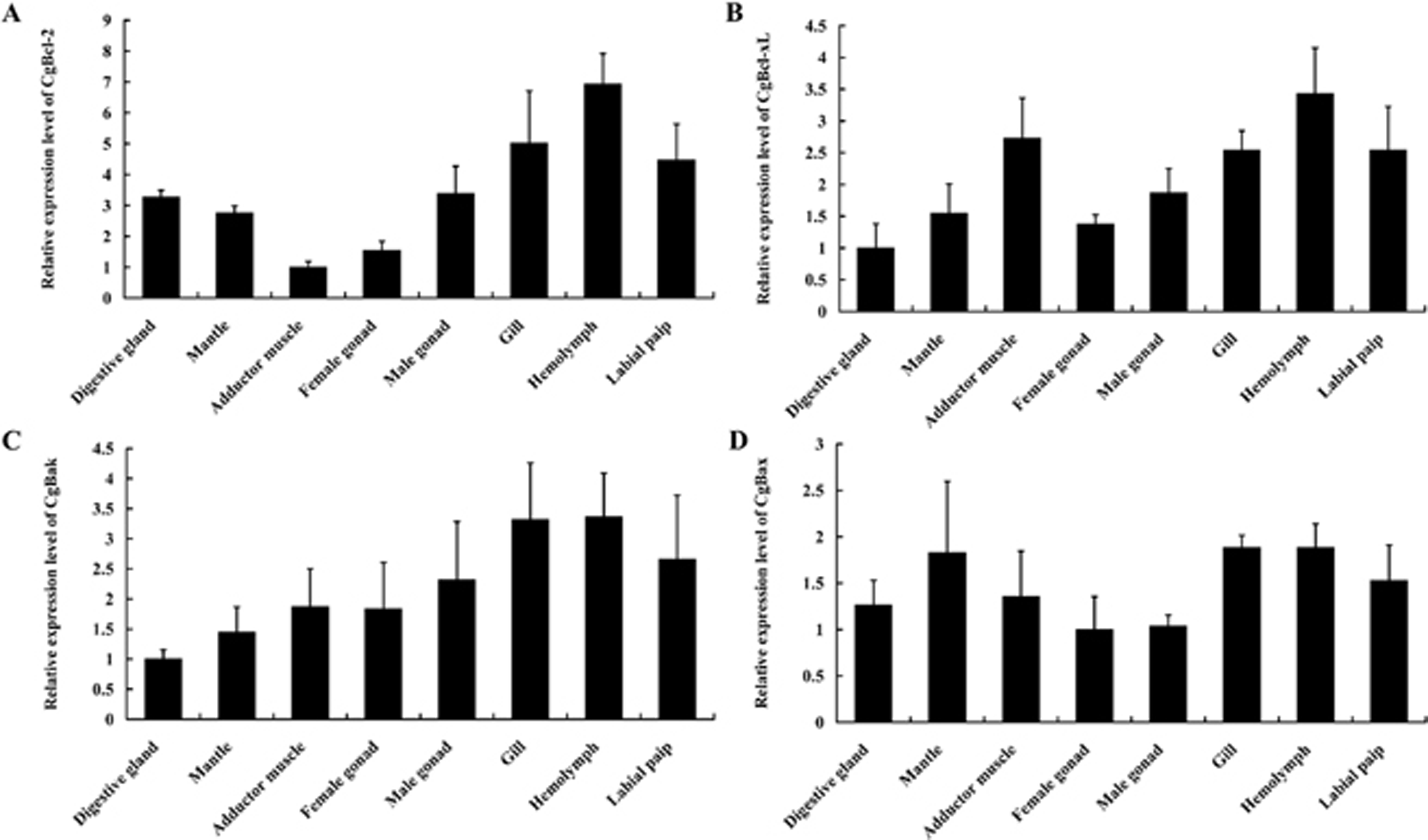

Supplement: Supplementary Figure 2 [file cddis2017307x2.tif]

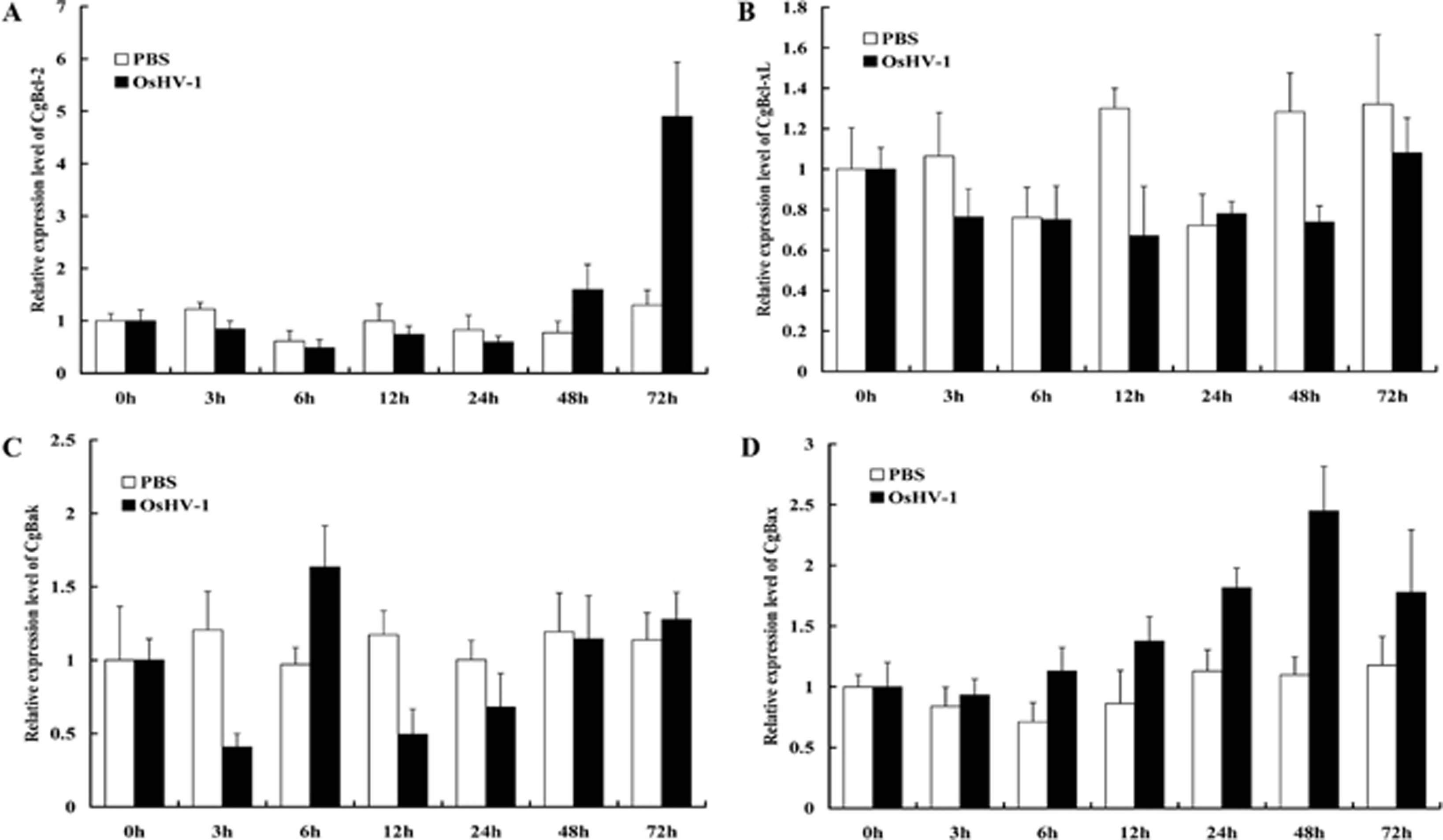

Supplement: Supplementary Figure 3 [file cddis2017307x3.tif]

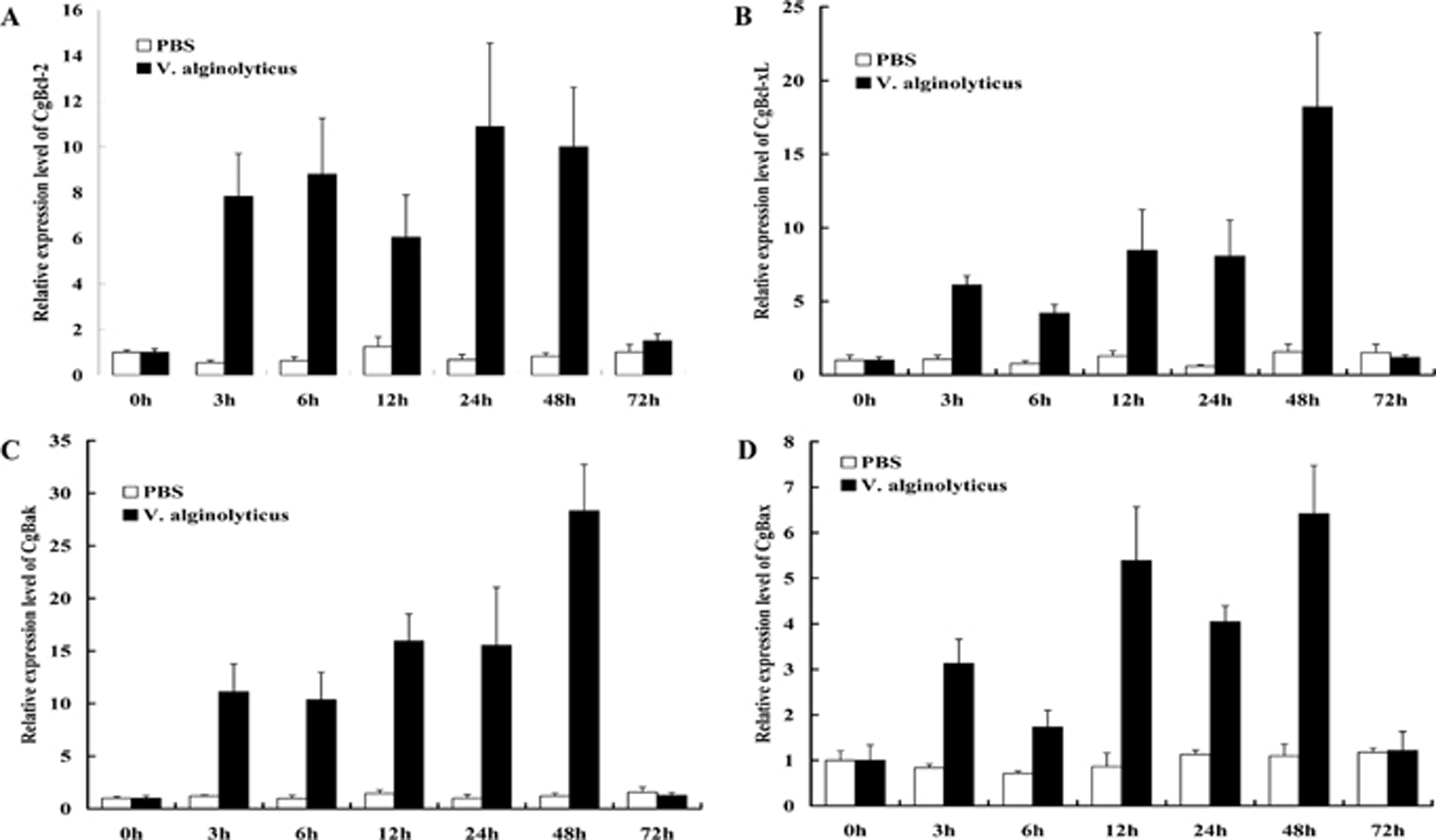

Supplement: Supplementary Figure 4 [file cddis2017307x4.tif]

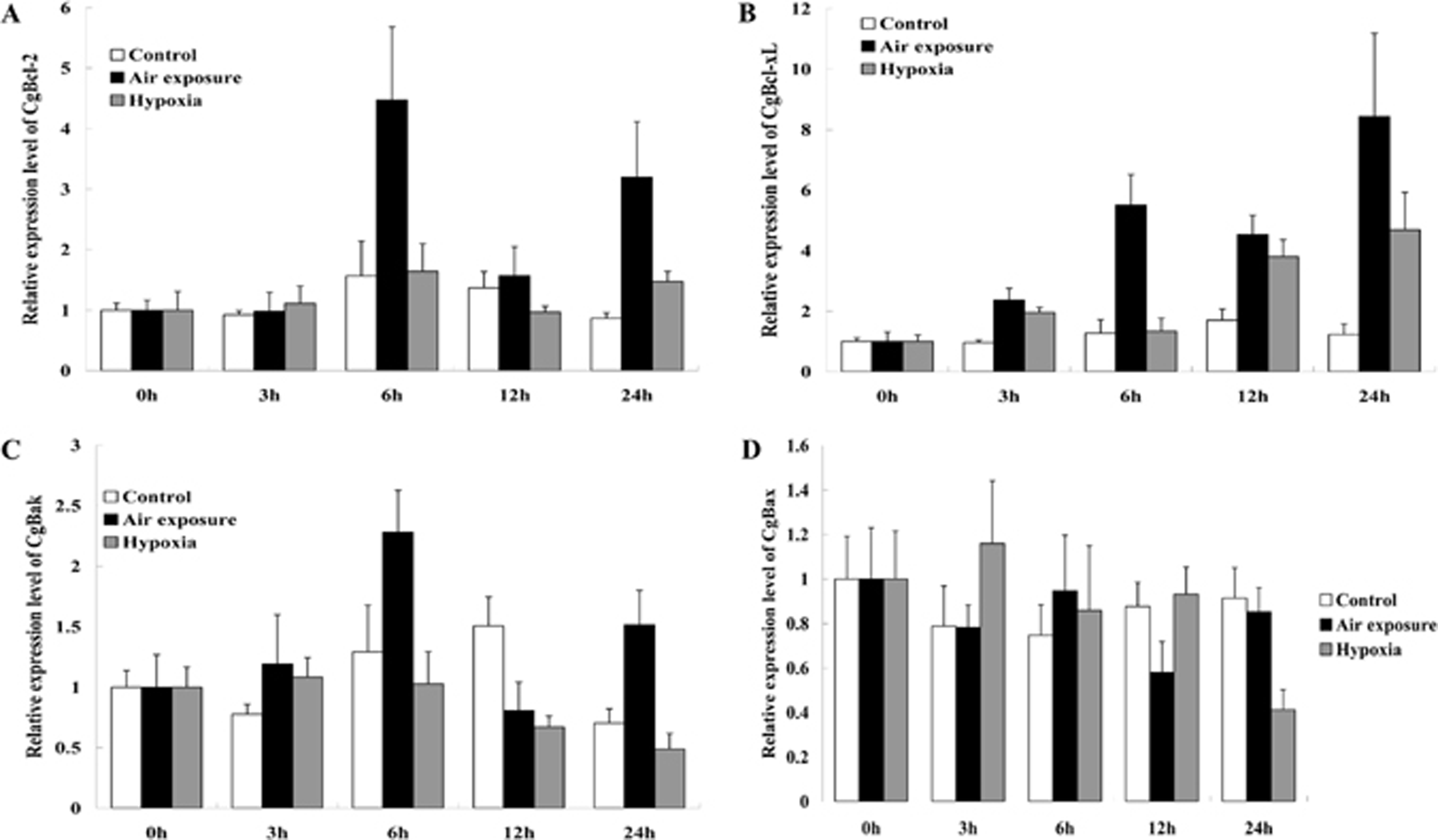

Supplement: Supplementary Figure 5 [file cddis2017307x5.tif]

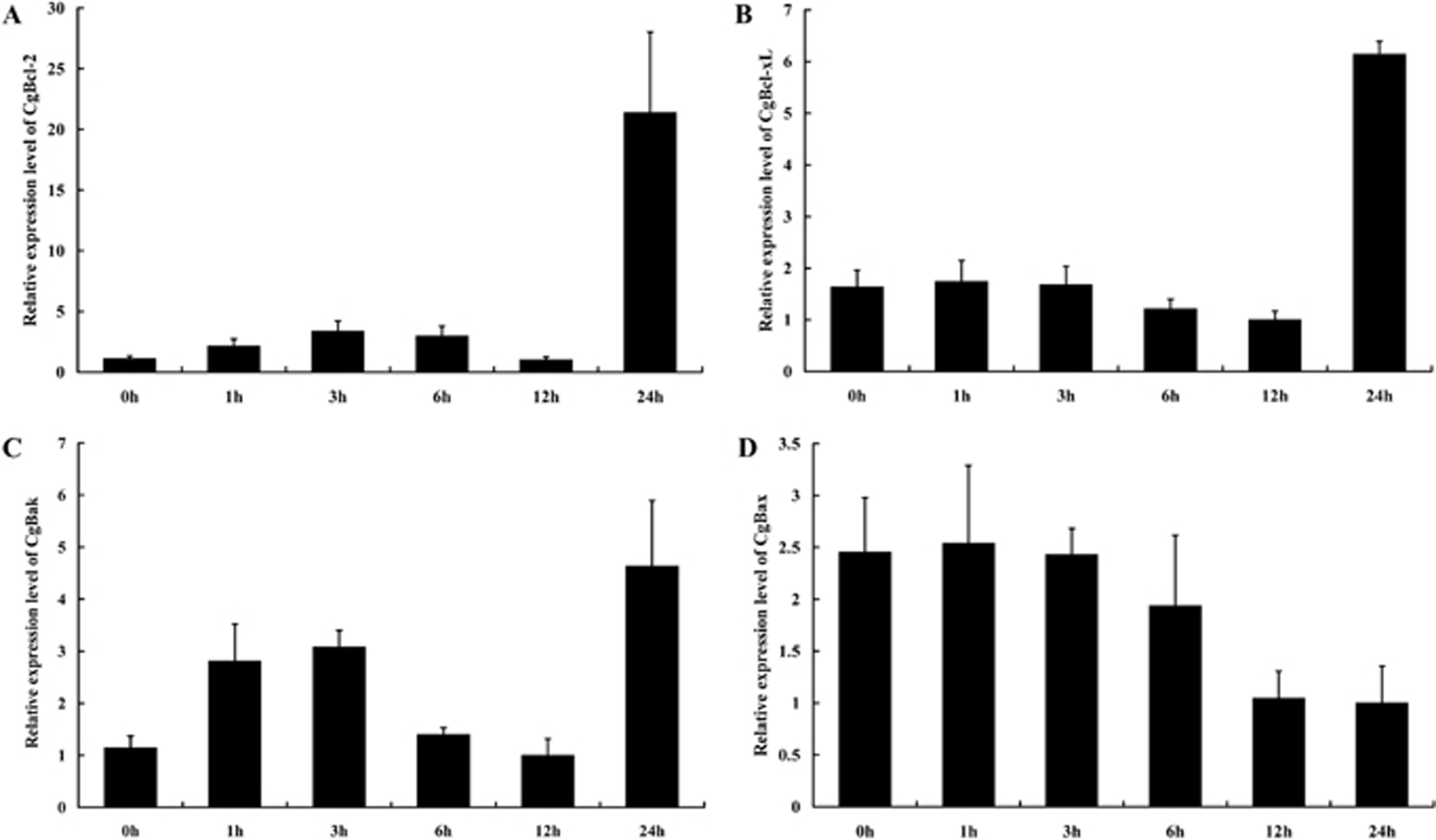

Supplement: Supplementary Figure 6 [file cddis2017307x6.tif]

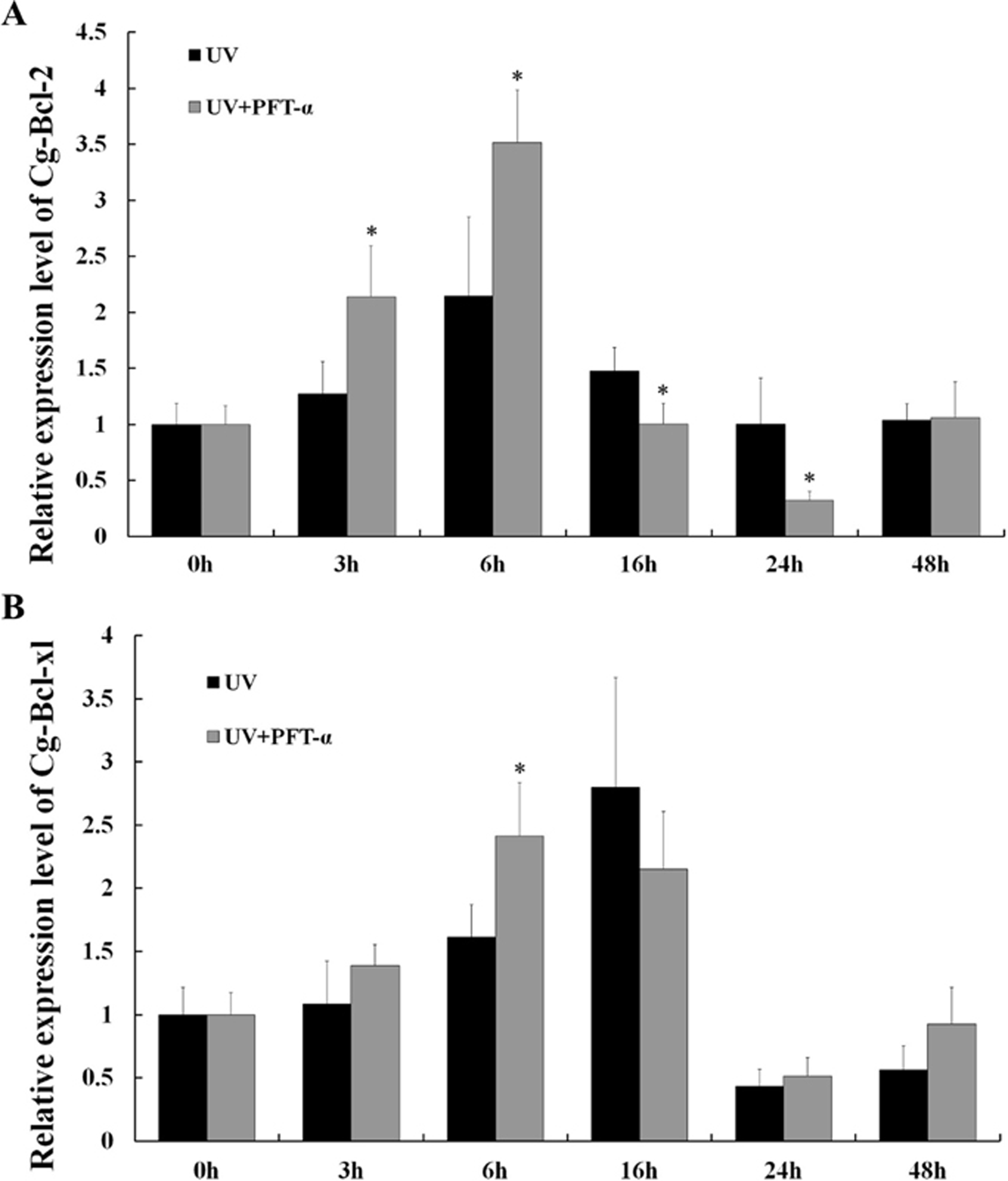

Supplement: Supplementary Figure 7 [file cddis2017307x7.tif]

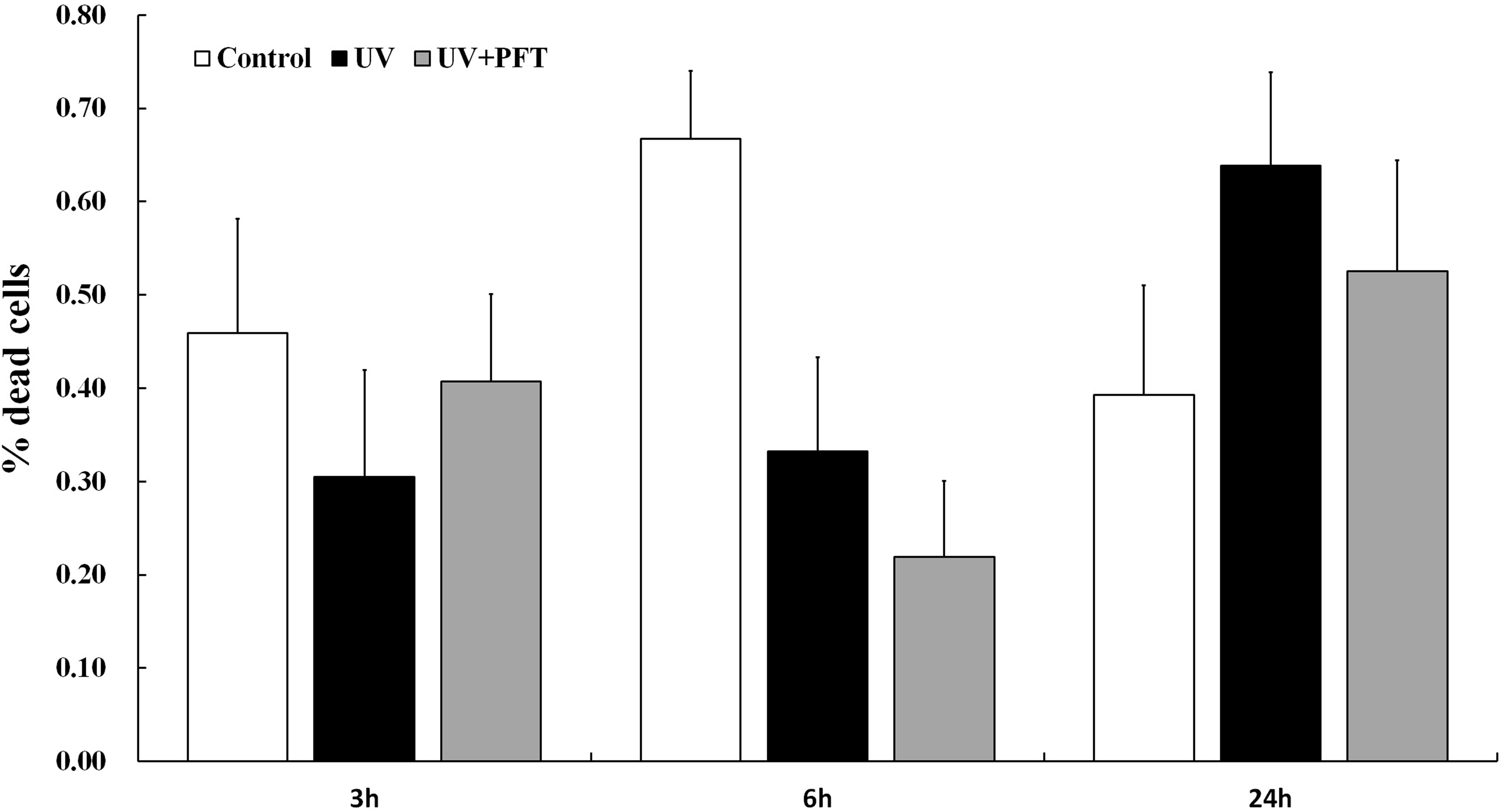

Supplement: Supplementary Figure 8 [file cddis2017307x8.tif]
