# Supplementary material for: Conservation and divergence of mitochondrial apoptosis pathway in the Pacific oyster, Crassostrea gigas
Source: Cell Death Dis. 2017 Jul 6;8(7):e2915–. doi: 10.1038/cddis.2017.307 (PMC5550854; doi:10.1038/cddis.2017.307)
Supplement: Supplementary Information [file cddis2017307x9.doc]

**Conservation and divergence of mitochondrial apoptosis pathway in the Pacific oyster, *Crassostrea gigas***

Yingxiang Li^1,2,3^, Linlin Zhang^1,2,3^, Tao Qu^1,2,3^, Xueying Tang^1,2,3,4^, Li Li*^1,2,3^, Guofan Zhang^1,2,3*^

^1^Key Laboratory of Experimental Marine Biology, Institute of Oceanology, Chinese Academy of Sciences

^2^Laboratory for Marine Biology and Biotechnology, Qingdao National Laboratory for Marine Science and Technology, Qingdao, China

^3^National & Local Joint Engineering Laboratory of Ecological Mariculture, Institute of Oceanology, Chinese Academy of Sciences, Qingdao, China

^4^University of Chinese Academy of Sciences, Beijing, China

*Corresponding authors: Li Li, Institute of Oceanology, Chinese Academy of Sciences, No.7 Nanhai Road, Qingdao 266071, China. Tel: +86 0532 82896728; Email: [lili@qdio.ac.cn](mailto:lili@qdio.ac.cn),

or Guofan Zhang, Institute of Oceanology, Chinese Academy of Sciences, No.7 Nanhai Road, Qingdao 266071, China. Tel: +86 0532 82898701; Email: [gfzhang@qdio.ac.cn](mailto:gfzhang@qdio.ac.cn)

**Running Title:** Mitochondrial Apoptosis in *Crassostrea gigas*

**Acknowledgements:** This research was supported by the National Natural Science Foundation of China (31530079), the Strategic Priority Research Program of "Western Pacific Ocean System: Structure, Dynamics and Consequences" (Grant No. XDA11000000), Technological Innovation Project Financially Supported by Qingdao National Laboratory for Marine Science and Technology (2015ASKJ02-03) and the Earmarked Fund for Modern Agro-industry Technology Research System (CARS-48).

**Abstract**

Apoptosis is considered a crucial part of the host defense system in oysters according to previous reports; however, the exact process by which this occurs remains unclear. Besides, mitochondrial apoptosis is the primary method of apoptosis in vertebrate cells, but has been poorly studied in invertebrates and is quite controversial. In this study, we investigated the molecular mechanism of mitochondrial apoptosis in the Pacific oyster *Crassostrea gigas*. Notably, we show that most key elements involved in the vertebrate mitochondrial apoptosis pathway—including mitochondrial outer membrane permeabilization (MOMP), cytochrome c release and caspase activation—are also present in *C. gigas*. In contrast, the lack of Bcl-2 homology 3 (BH3)-only subfamily members and apoptotic protease activating factor-1 (APAF-1) protein revealed evolutionary diversity from other phyla. Our results support that mitochondrial apoptosis in animals predates the emergence of vertebrates, but suggest that an unexpectedly diverse mitochondrial apoptosis pathway may exist in invertebrates. In addition, our work provided new clues for an improved understanding of how bivalve acclimate themselves to an inconstant environment.

**Introduction**

Oysters live in estuarine and intertidal zones and are constantly exposed to fluctuating temperatures, variable salinity, toxic metals, and desiccation because of their sessile behavior.^1^ Apoptosis is involved in the response to various stressors and an important host defense mechanism in oysters. For instance, apoptosis participates in the oyster’s defense to *Perkinsus marinus*, an intracellular protozoan that can cause lethal infection.^2^ Others have also shown that exposure to cadmium and copper elevates apoptosis levels in oysters.^3,4^ The recent application of high-throughput sequencing in marine biology studies has revealed the extensive involvement of apoptosis-related genes in various stress responses, including those to virus,^5^ air exposure,^1^ hyperthermia,^6^ and low salinity.^7^ However, no studies have focused on elucidating the molecular pathways regulating apoptosis in oyster and the exact physiological mechanisms remain vague, hindering our understanding of how this sedentary animal adapts to its fickle environment.

The mitochondrial or intrinsic apoptosis pathway is characterized by the participation of mitochondria in apoptotic signaling and has been extensively described in vertebrates, including mammals^8,9^ and amphibians.^10,11^ Several genes homologous to mammalian apoptosis effectors have been identified in invertebrates in recent years,^12-15^ seemingly indicative of the existence of a mitochondrial apoptosis pathway in these species.

In comparison to the breadth of data on the molecular physiology of mammalian mitochondrial apoptosis, relatively few mechanisms in invertebrates have been elucidated.^16-19^ Nevertheless, current evidence suggests an increased diversity of pathway effectors in these species. For instance, mitochondrial outer membrane permeabilization (MOMP) and cytochrome *c* release—hallmarks of vertebrate mitochondrial apoptosis—played no role in mitochondrial apoptosis in the nematode *Caenorhabditis elegans*.^16^ More specifically, cell death protein 4 (CED-4)—the *C. elegans* APAF-1 homolog—lacks the WD domain responsible for its interaction with cytochrome *c*.^16^ Similarly, while the *Drosophila* APAF-1-related killer (ARK) homolog contains this region,^17^ no biochemical evidence exists on the involvement of cytochrome *c* in ARK activation and induction of the apoptosis cascade.^20^ As such, additional work is needed to determine the extent of this diversity in invertebrate mitochondrial apoptosis and further understand the pathway’s evolution in animals.

Cursory studies on bivalve apoptosis have been performed,^21-25^ yet the exact mechanism of mitochondrial apoptosis remains unclear. This study has three specific goals and therefore includes three aspects of work. Firstly, to investigate whether MOMP and cytochrome *c* are involved in mitochondrial apoptosis of oyster, we tested the mitochondrial membrane potential and subcellular distribution of cytochrome *c* in UV irradiated oyster hemocytes. Secondly, to examine whether members of oyster caspase family regulate mitochondrial apoptosis, activities of caspase 9 and caspase 3 in hemocytes and cytosolic extracts of oyster upon distinct treatments were measured. Thirdly, to elucidate the regulatory roles of Bcl-2 family and p53 in mitochondrial apoptosis pathway of oyster, multiple functional assays were performed. Given that oyster cell lines are not widely available currently, mammalian and yeast cells were used in several assays as molecular tools, which is a typical practice for species without established cell lines.^18,19,26^ Our work significantly improves mechanism understanding of mitochondrial apoptosis pathway in the Pacific oyster and expands our knowledge of evolutionary diversity of apoptosis system in invertebrates.

**Results**

**UV irradiation results in loss of mitochondrial membrane potential (MMP) and cytochrome *c* release in *C. gigas*.**

UV irradiation is an effective apoptosis-inducing factor in many species, including bivalves.^25,27-29^ Thus, we irradiated *C. gigas* hemocytes with UV light and found a markedly higher percentage of apoptotic cells when compared to non-irradiated controls (Fig. 1A). Additionally, transmission electron microscopy analysis showed the presence of apoptotic cells under UV light irradiation (S1 Fig), further indicating that UV irradiation was an efficacious apoptosis-inducing factor in *C. gigas*.

The loss of MMP (ΔΨm) is an indication of early stage of apoptosis, as it is associated with mitochondrial outer membrane permeabilization in vertebrates.^30^ Thus, we examined MMP with JC-1 assays to determine whether mitochondrial apoptosis in Pacific oyster involved an increase in mitochondrial outer membrane permeability. Notably, irradiated hemocytes exhibited a significant decrease in MMP at 6 h post-irradiation (hpi), which further decreased at 9 hpi and 24 hpi (Fig. 1B), indicating the existence of an MOMP-like process in UV irradiation-induced apoptosis in *C. gigas*.

Vertebrate MOMP results in cytochrome *c* release and triggers mitochondria-mediated apoptosis. To determine if cytochrome *c* release occurred in *C. gigas* cells, UV-irradiated hemocytes were subjected to cytosolic/mitochondrial subcellular fractionation at 24 hpi and the presence of cytochrome *c* was assessed in each fraction. Western blot analysis revealed that cytochrome *c* was found in the cytosolic protein fraction of irradiated cells, but undetectable in that of untreated cells (Fig. 1C), suggesting that UV irradiation induced release of cytochrome *c* from mitochondria to the cytosol.

**Cytochrome *c* activates caspase 9 and caspase 3 activity.**

We tested the activities of effector caspase 9 and executioner caspase 3 in oyster hemocytes following UV irradiation and found remarkable elevations in the activity levels of both caspases at 20 hpi (Fig. 2A and 2B), indicating the involvement of *C. gigas* (Cg)-caspase 9 and Cg-caspase 3 in irradiation-induced apoptosis. We then incubated cytosolic extracts with cytochrome *c* purified from equine hearts or *C. gigas* (Cg-Cyt *c*). Notably, both Cg-caspase 9 and Cg-caspase 3 activity was significantly increased in the presence of cytochrome *c* from either source (Fig. 2C and 2D). Further, the addition of exogenous dATP enhanced the cytochrome *c*-mediated-activation of both caspases (Fig. 2C and 2D).

To determine whether Cg-caspase 3 activity was dependent on that of Cg-caspase 9, oyster cytosolic extracts were pretreated with the caspase 9 inhibitor Z-LEHD-FMK before the addition of Cg-Cyt *c*. As expected, Cg-caspase 9 activity was substantially decreased significantly with the addition of Z-LEHD-FMK (Fig. 2C). Moreover, irradiated hemocytes pretreated with Z-LEHD-FMK showed a markedly lower activity for both Cg-caspase 3 and Cg-caspase 9 (Fig. 2E), demonstrating that the functions of these caspases in *C. gigas* were comparable to their vertebrate counterparts.

**Identification and expression analysis of Bcl-2 relatives in the Pacific oyster.**

B-cell lymphoma **(**Bcl)-2 protein family members are known regulators of mitochondrial apoptosis. We identified seven candidate Bcl-2 homologs in the Pacific oyster by querying the annotated genome database, but none belonged to the pro-apoptotic “BH3-only” subfamily. Nevertheless, we performed rapid-amplification of cDNA ends (RACE) or PCR to amplify the entire coding sequence and subsequently validated their genetic sequences.

To study the functional significance of oyster Bcl-2 family members, we selected four candidate genes out of the seven, as they were more closely related to human Bcl-2 family members with regard to the domain architecture. We designated them as *Cg-Bcl-2*, *Cg-Bcl-xl*, *Cg-Bak*, and *Cg-Bax* based on their homology to human relatives. All of the four candidates contained BH1, BH2, and BH3 domains, while only Cg-Bcl-2 had a BH4 domain (Fig. 3A). In addition, Cg-Bak and Cg-Bax harbored a transmembrane domain absent in Cg-Bcl-2 and Cg-Bcl-xl (Fig. 3A). An amino acid sequence-based phylogenetic analysis showed that Cg-Bcl-2 and Cg-Bcl-xl clustered into the anti-apoptotic protein category, whereas Cg-Bak and Cg-Bax classified as pro-apoptotic proteins (Fig. 3B).

Quantitative PCR (qPCR) analysis was performed to investigate the expression pattern of oyster Bcl-2 family genes in different tissues and under various environmental stresses. All the four genes displayed relatively high expression in gill and hemolymph (S2 Fig). Moreover, infection with ostreid herpesvirus 1 (OsHV-1, a common virus that can cause mass mortality of oyster) or *Vibrio alginolyticus*, as well air exposure and heat shock, dramatically altered the expressions of these Bcl-2 relatives (S3 Fig, S4 Fig, S5 Fig, and S6 Fig).

***C. gigas* Bcl-2 family members regulate apoptosis signaling.**

We first examined the contributions of the Pacific oyster Bcl-2 candidate homologs in cell death in the yeast *Saccharomy**ces cerevisiae*, as Bax and Bak from human^31^ and *Schistosoma mediterranea*^18^ can trigger cell death in yeast according to previous reports. Significantly, we found that both Cg-Bak and Cg-Bax induced yeast cell death (Fig. 4A); however, coexpression of either Cg-Bcl-2 or human Bcl-2 rescued yeast from Cg-Bak- or Cg-Bax-induced lethality (Fig. 4A).

To further determine the roles of oyster Bcl-2 family members in apoptosis, we overexpressed Cg-Bak or Cg-Bax in HEK293T cells. Interestingly, neither Cg-Bak or Cg-Bax altered caspase 3 activity in these cells, nor did Cg-Bcl-xl or Cg-Bcl-2 (data not shown). However, H_2_O_2_-induced oxidative stress effectively stimulated caspase 3 activity level in HEK293T cells, which was markedly increased in counterparts with Cg-Bak or Cg-Bax overexpression (Fig. 4B and 4C). Additionally, the co-expression of Cg-Bcl-2 or Cg-Bcl-xl with Cg-Bak or Cg-Bax significantly attenuated the caspase 3 activity in H_2_O_2_-treated cells when compared to treated controls with Cg-Bak or Cg-Bax expression alone (Fig. 4B and 4C).

We then analyzed the expression of the four Bcl-2 family genes following UV irradiation and found a remarkable induction of all four candidates (Fig. 4D), indicative of their involvement in apoptosis regulation. We performed siRNA assays on *Cg-Bak* and *Cg-Bax*, which effectively suppressed their expression (Fig. 4E and 4F). Subsequently, we irradiated *Cg-Bak*-silenced, *Cg-Bax*-silenced, and scrambled siRNA-treated hemocytes and measured apoptosis at 3 and 24 hpi, which revealed that *Cg-Bak*-silenced and *Cg-Bax*-silenced hemocytes were both significantly more resistant to UV-induced apoptosis than their scrambled siRNA-treated counterparts at 24 hpi (Fig. 4G and 4H).

**Cg-Bak and Cg-Bax translocate to the mitochondria and induce cytochrome *c* release during apoptosis.**

To understand how the pro-apoptotic oyster Bcl-2 family proteins regulate mitochondrial apoptosis, we studied the subcellular localization of Cg-Bak and Cg-Bax in HeLa cells. Fluorescence microscopy showed that both Cg-Bak and Cg-Bax mainly localized throughout the cytoplasm of normal cells, but translocated to the mitochondria in a subset of cells following UV irradiation. (Fig. 5A and 5B). This data was also validated in subcellular fractionation experiments in HEK293T cells with Cg-Bak and Cg-Bax overexpression (Fig. 5C).

To ascertain whether the mitochondrial translocation of Cg-Bak and Cg-Bax was associated with their regulatory roles in apoptosis, we examined the impact of purified Cg-Bak and Cg-Bax protein on isolated oyster mitochondria. When control mitochondria were incubated in storage buffer and centrifuged, cytochrome *c* could only be detected in the precipitate; however, incubation with CaCl_2_ resulted in detectable cytochrome *c* only in the supernatant (Fig. 5D). Subsequent analyses showed that cytochrome *c* was detectable in both the supernatant and precipitate when mitochondria were incubated with recombinant Cg-Bak or Cg-Bax (Fig. 5D).

**Anti-apoptotic Bcl-2 family proteins directly bind Cg-Bak and Cg-Bax.**

In mammals, anti-apoptotic Bcl-2 subfamily members interact with their pro-apoptotic counterparts, thereby inhibiting the activities of the latter.^32^ Thus, we used a yeast two-hybrid system to determine if this regulatory interaction also occurred with *C. gigas* homologs. Notably, both Cg-Bcl-2 and Cg-Bcl-xl were found to interact with pro-apoptotic Cg-Bak and Cg-Bax (Fig. 6A). A series of truncated mutants were constructed to identify the domain essential for mediating these interactions (Fig. 6B). Results of yeast two-hybrid assays with the truncated mutants indicated that the BH3 domain facilitated the interactions between oyster Bcl-2 family members (Fig. 6C), which was subsequently confirmed with co-immunoprecipitation (co-IP) assays. (Fig. 6D and 6E).

***C. gigas* Cg-p53 is involved in mitochondrial apoptosis.**

Despite the application of high-throughput sequencing technologies, a BH3-only homolog is yet to be discovered in mollusk.^21,33,34^ This absence led us to identify other regulators of mitochondrial apoptosis upstream of the Bcl-2 homologs. We first considered the oyster p53 homolog Cg-p53 as a candidate, since p53 is known to regulate Bcl-2 family activities in mammals.^35^ Our preliminary analysis of *Cg-p53* expression in oyster hemocyte showed a substantial upregulation in response to UV irradiation (Fig. 7A). We then treated the hemocytes with the p53 inhibitor pifithrin-α and validated its effect of PFT-α on *Cg-p53* expression (Fig. 7B). Moreover, analysis of UV-irradiation-induced apoptosis in PFT-α-treated hemocytes revealed that p53 inhibition substantially increased their apoptotic resistance at 3 hpi and 6 hpi, but not at 24 hpi, when compared to untreated controls (Fig. 7C). This is consistent with an increase of *Cg-Bcl-2* and *Cg-Bcl-xl* levels (S7 Fig) together with the delay in *Cg-Bak* activation, thus suggesting that the absence of p53 could promote apoptosis resistance through Cg-Bcl-2 and Cg-Bcl-xl.

**Cg-p53 regulates the Bcl-2 family in mitochondrial apoptosis pathway of Pacific oyster.**

Dual-luciferase reporter assays were used to monitor Cg-p53 transcriptional activity. The ~2 kb region upstream of the *Cg-Bak*, *Cg-Bax*, *Cg-Bcl-2*, and *Cg-Bcl-xl* start codons were considered as potential promoter regions and used to construct the *Cg-Bak*-Luc*, Cg-Bax*-Luc*, Cg-Bcl-2*-Luc, *and Cg-Bcl-xl*-Luc plasmids (Fig. 8A). In preliminary experiments with HEK293T cells, Cg-p53 elicited the transcription from *Cg-Bak-Luc* and *Cg-Bax-Luc* promoters whereas it repressed the transcription from *Cg-Bcl-2-Luc* and *Cg-Bcl-xl-Luc* promoters, all in a dose-dependent manner (Fig. 8B, 8C, 8D, and 8E).

To determine whether Cg-p53 could regulate *Cg-Bak* and *Cg-Bax* in oyster cells, the expression patterns of *Cg-Bak* and *Cg-Bax* in PFT-α treated hemocytes after UV light irradiation were determined. The qPCR data showed that PFT-α treatment significantly delayed the activation of *Cg-Bak* expression upon irradiation, compared with that in untreated hemocytes (Fig. 8F), indicating that Cg-p53 transactivates *Cg-Bak* in *C. gigas*. For *Cg-Bax* however, the timing of activation is mostly unchanged in both PFT-α-treated and non-treated hemocytes (Fig 8G).

Considering that mammalian p53 regulates the activities of both anti-apoptotic and pro-apoptotic Bcl-2 family proteins through a transcription-independent way (protein-protein interaction), Cg-p53 may also bind to oyster Bcl-2 family members. This could serve as a plausible explanation for the unexpected absence of *Cg-Bax* promoter activity in PFT-α treated irradiated hemocytes. Thus, we performed a co-IP assay on Cg-p53 in HEK293T cells with the four Bcl-2 family proteins of *C. gigas*, yielding positive results; hence, probable interactions exist between the proteins in question (Fig. 8H).

**Discussion**

The mechanisms of invertebrate mitochondrial apoptosis are unclear. Current evidence demonstrates that neither MOMP and cytochrome *c* release regulate mitochondrial apoptosis in *Drosophila melanogaster* and *Caenorhabditis elegans*; however, these events are essential for that in *Schmidtea mediterranea*.^36-39^ Therefore, further analysis should be carried out in other invertebrate species to elucidate the evolutionary physiology of mitochondrial apoptosis in invertebrates. Herein, we demonstrated the existence of MOMP and cytochrome *c* release in mitochondrial apoptosis of *C. gigas*. These results confirmed previous speculation that MOMP and cytochrome *c* release emerged prior to that of deuterostomes, but somehow missed in ecdysozoa species—at least with respect to *Drosophila melanogaster* and *Caenorhabditis elegans*.^20^

Caspase 9 and caspase 3 are proteases with vital roles in mitochondrial apoptosis. In this study, we found that both Cg-caspase 9 and Cg-caspase 3 were involved in mitochondrial apoptosis in *C. gigas*, with Cg-caspase 9 likely acting as an upstream activator of Cg-caspase 3—consistent with their roles in vertebrates.^40^ However, the oyster APAF-1 homolog lacks the caspase recruitment domain (CARD domain) and only possesses the cytochrome *c-*interacting WD domain. In fact, no CARD-domain-containing APAF-1 homolog has been identified in the Mollusca phylum to date, supporting the existence of a distinct mechanism of caspase 9 activation. Interestingly, the *C. elegans* APAF-1 homolog contains a CARD domain, but no WD domain,^41^ indicative of pathway diversity throughout invertebrates.

Bcl-2 family proteins are central regulators of mitochondrial apoptosis. In this study, we identified the existence of oyster Bcl-2 family genes and demonstrated their regulatory roles in apoptosis. Notably, both Cg-Bak and Cg-Bax exhibited pro-apoptotic effects in yeast, consistent with that previously observed with their human^31^ and *Schistosoma mediterranea* homologs.^18^ The present work sought to define the functional significance of Cg-Bak and Cg-Bax in mitochondrial apoptosis, and revealed that both factors translocate from the cytoplasm to mitochondria in mammalian cells in response to UV irradiation. This result indicates that while Cg-Bax likely functions in a similar manner to its mammalian counterpart,^42,43^ Cg-Bak may utilize an independent mechanisms as mammalian Bak mainly localizes to the mitochondrial outer membrane in healthy cells.^44^ However, the inconsistencies in Cg-Bak and mammalian Bak subcellular localization might be the result of structural differences or the misfolding of Cg-Bak in mammalian cells. We were unable to examine the distribution of these proteins in oyster cells due to the lack of suitable antibodies. Despite this, the result herein could still imply the association of the proteins with mitochondria during apoptosis. In fact, our previous study demonstrates a potential interaction between Cg-Bak and the mitochondrial outer membrane porin Cg-VDAC2.^45^ Nevertheless, our results support that Cg-Bak and Cg-Bax induce cytochrome *c* release, as observed with mammalian Bak and Bax. Moreover, our study also revealed that the interaction of antiapoptotic proteins (Cg-Bcl-2 and Cg-Bcl-xl) and proapoptotic proteins (Cg-Bak and Cg-Bax) in *C. gigas* was mediated by the BH3 domain, consistent with vertebrates.^46,47^ Additionally, while *Cg-Bak* was found to be transcriptional activated by Cg-p53, *Cg-Bax* seemed to be more loosely regulated, although its promoter could be activated by Cg-p53 in mammalian cells. This might not result from the regulation by *Cg-Bcl-2* or *Cg-Bcl-xl*, as the anti-apoptotic Bcl-2 family members were both negatively regulated by Cg-p53. Therefore, it will be an interesting work to clarify the mechanisms regulating *Cg-Bax*. Collectively, the apoptotic functions of the oyster Bcl-2 family proteins investigated here are fairly conserved with vertebrate counterparts, as was the transcriptional activity of Cg-p53.^48^

Significantly, we failed to discover a BH3-only homolog in *C. gigas*, nor has one been identified in the entire Mollusca phylum.^33,34^ BH3-only proteins participate in the apoptotic response to various stressors.^46^ As such, the absence of a BH3-only homolog may imply a less complex regulatory network in *C. gigas,* or in all Mollusca species. A simpler network might expedite the process of apoptosis, particularly in species that lack adaptive immunity^49^ and extrinsic apoptosis.^50^ Alternatively, it might also indicate a less stable apoptotic regulatory system. Either way, the impact of missing BH3-only proteins on apoptosis and the environmental adaptation mechanism of *C. gigas* and mollusks is of future interest.

Apoptosis is a crucial host defense mechanism.^51^ Our confirmation of mitochondrial apoptosis in *C. gigas* and the regulatory functions of oyster Bcl-2 family members, along with the stimulus-induced expression differences of the oyster Bcl-2 family genes under various stresses, support the extensive involvement of mitochondrial apoptosis in the environmental adaptation process of *C. gigas*. However, much effort will be needed to clarify the exact role of mitochondrial apoptosis in the response to various stressors.

In conclusion, the present study characterized the conservation and divergence of mitochondrial apoptosis in the Pacific oyster *C. gigas*. However, it is important to point out that many functional experiments were conducted in yeast or human cell lines as molecular tools in this study given the limitation of our experimental system. The limitation that is general to many non-model organisms is that cell lines and cell line-based research such as gene overexpression, protein-protein interaction is still challenging. However, despite these limitations, we found most events in *C. gigas* mitochondrial apoptosis displayed great similarity to that in vertebrate and *Schmidtea mediterranea* including MOMP and cytochrome c release. On the other hand, the absence of BH3-only proteins and the lack of a domain-intact APAF-1 homolog in Pacific oyster were indicative of pathway diversity in invertebrates. Continued investigation in other invertebrates will aid in our understanding of mitochondrial apoptosis pathway evolution.

**Materials and Methods**

**Chemicals, plasmids, small interfering RNAs, and antibodies.** H_2_O_2_ was purchased from Sangon (Shanghai, China). Cytochrome c from equine heart was purchased from Solarbio (Beijing, China) and *C. gigas* cytochrome c was synthesized by Chemgen Biotechnology (Shanghai, China). Deoxyadenosine triphosphate (dATP) was obtained from Tiangen (Beijing, China). The Z-LEHD-FMK and PFT-α inhibitors were purchased from BioVision (Milpitas, CA, USA) and Beyotime (Jiangsu, China), respectively. The pGADT7, pGBKT7, and pEGFP-N1 plasmids were purchased from Takara (Shiga, Japan). pCMV-N-Myc and pCMV-N-FLAG plasmids were obtained from Beyotime (Jiangsu, China) and the pYES2/CT from Invitrogen (Carlsbad, CA, USA). The pRL-TK and pGL3-basic plasmids were both from Promega (Madison, MI, USA). Small-interfering RNAs targeting *Cg-Bak* (Cg-Bak1 sense: GGACGUGAUCAGGAACUUUTT, anti-sense: AAAGUUCCUGAUCACGUCCTT; CgBak2 sense: GGCGAUAUGCCGAUGAAUUTT, anti-sense: AAUUCAUCGGCAUAUCGCCTT; CgBak3 sense: GGUCAUUGUCAGUCAUGUUTT, anti-sense: AACAUGACUGACAAUGACCTT; scrambled negative control siRNA sense: UUCUCCGAACGUGUCACGUdTdT, anti-sense: ACGUGACACGUUCGGAGAAdTdT) and *Cg-Bax* (Cg-Bax1 sense: GCAGAGCUUUACGUUGCAUTT, anti-sense: AUGCAACGUAAAGCUCUGCTT; Cg-Bax2 sense: CCUUUGGAAGGCACUCAAATT, anti-sense: UUUGAGUGCCUUCCAAAGGTT; Cg-Bax3 sense: GCAGAGUGGGAUGUCUAUUTT, anti-sense: AAUAGACAUCCCACUCUGCTT) were obtained from GenePharma (Shanghai, China). Antibodies against FLAG and EGFP were purchased from Sigma (St. Louis, MO, USA). Myc antibody was from Roche (Basel, Switzerland). Antibody against cytochrome c was synthesized by Chemgen Biotechnology (Shanghai, China). Peroxidase-labeled mouse IgG secondary antibody was purchased from KPL (Gaithersburg, MD, USA).

**Oyster and primary hemocyte culture.** Pacific oysters averaging 7 cm in shell height were obtained from a local farmer in Qingdao, China, and acclimatized in tanks with 20 ± 0.5 °C, pH 8 ± 0.3 seawater. Primary hemocyte culture was performed as previously described with slight modifications.^52^ Briefly, hemocytes were withdrawn from the pericardial cavity using a sterile 21-gauge needle attached to a 1 mL syringe containing 0.1-0.2 mL ice-cold filtered sterile seawater (FSW) supplemented with 2 g/L glucose. The cells were plated into 6-well plates, adjusted to approximate 2 × 10^6^ per well, and allowed to adhere for 30 min at room temperature. Hemolymph then was removed and Leibovitz L-15 medium (Sigma, Louis, MO, USA) supplemented with additives^52^ (including 0.54 g/L KCl, 20.2 g/L NaCl, 0.6 g/L CaCl_2_, 3.9 g/L MgCl_2_, 1 g/L MgSO_4_, 20.8 g/L glucose, 220 μg/mL streptomycin, 100 μg/mL gentamicin, 100 μg/mL penicillin, and 0.1 μg/mL amphotericin B) was added to suspend the cells before culturing at 18 °C.

**Viral challenge, vibrio challenge, heat shock, air exposure, and UV irradiation.** Viruses were obtained as previously described.^53^ Live *Vibrio alginolyticus* were suspended with sterilized PBS and adjusted to 5 × 10^7^ cells/mL. Before challenge, a small hole was made, using a sand-saw, on the edge of the oysters’ shells to accommodate the injection. Three days later, 0.1 mL viral homogenate, the *Vibrio alginolyticus* suspension or PBS was injected into the oysters. Six individuals from each challenge group were randomly sampled at 0, 3, 6, 12, 24, 48, and 72 h after injection and the hemocytes were collected. For heat shock treatment, oysters were placed in tanks with 35 °C seawater for 1 h, transferred back to 25 ± 0.5 °C seawater, and then, six individuals from each group (treated and untreated) were sampled 0, 1, 3, 6, 12, and 24 h later. For air exposure experiment, oysters were placed in a tank without seawater at 20 ± 1 °C and then tissues were collected from six individuals in each group (treated and untreated) at 1, 3, 5, 7, 9, 10, and 11 days after exposure. For UV irradiation, cell culture medium was reduced to 500  μL prior to irradiation. Hemocytes were then exposed to UV light (15W, Philips, Netherlands) for 20 min, the media volume restored to 1 mL, and cells were sampled at distinct time points based on the analysis. Hemocytes from six independent wells were sampled as experimental replicates at each time point.

**Transmission electron microscopy (TEM).** TEM analysis of oyster hemocytes was performed following a previous study.^29^ Hemocyte suspensions (1 × 10^6^ cells) were centrifuged at 500 × *g* for 8 min at 4°C, and the supernatant was discarded. Samples were fixed in 3% glutaraldehyde solution for 1 day at 4°C. After 3 washes with 0.4 M cacodylate buffer, the cells were post-fixed with a solution of 1% osmium tetroxide for 1 h at 4°C. Then, the cells were washed twice in 0.4 M cacodylate buffer. After dehydration in successive baths of ethanol and two baths of propylene oxide, samples were progressively impregnated and embedded in Epon. After polymerization at 60°C, semi-thin sections were cut to 1 mm thickness for quality control and then to 80-85 nm for examination on a Leica Ultracut (EM UC6), floated onto copper EM grids, and stained with uracil acetate/Fahmys lead citrate. The sections were examined using a transmission electron microscope (H-7000, Hitachi, Japan).

**Real-time PCR analysis.** Total RNA from tissues and cells were extracted using TRIzol (Invitrogen, Carlsbad, CA, USA) following the manufacturer’s instructions. RNA integrity was assessed using agarose gel electrophoresis, and purity was estimating according to the A260/280 absorption ratio measured using a NanoDrop 2000 Spectrophotometer (Thermo Fisher Scientific, Sunnyvale, CA, USA). cDNA was synthesized by reverse transcription with the PrimeScript RT reagent kit with gDNA Eraser (TaKaRa, Shiga, Japan). Real-time PCR was carried out on an ABI 7500 Fast Real-Time PCR System (Applied Biosystems, Foster City, CA, USA) using 2X SYBR Ex Taq mix (TaKaRa, Shiga, Japan) according to the manufacturer’s protocol. Cg-GAPDH was used as an internal control for expression analysis in response to OsHV-1, heat shock and air exposure.^23,45^ Cg-β-actin was used as an internal control in UV light irradiation analyses.^25^

**Mammal cell cultures and transfection.** HEK293T cells (ATCC, Manassas, VA, USA) were cultured in high-glucose Dulbecco’s Modified Eagle’s Medium (DMEM) (HyClone, Logan, UT, USA). HeLa cells were cultured in modified Roswell Park Memorial Institute (RPMI)-1640 medium (HyClone, Logan, UT, USA). Both media were supplemented with 10% fetal bovine serum (HyClone, Logan, UT, USA), penicillin (100 U/mL), and streptomycin (100 U/mL). Cells were maintained at 37 °C in a humidified atmosphere of 5% CO_2_. Plasmid transient transfection was performed with Lipofectamine 3000 (Invitrogen, Carlsbad, CA, USA) according to the manufacturer’s protocol.

**Yeast Death Assays.** pYES2/CT constructs carrying distinct fragments with or without pADH with the indicated inserts were transformed into *S. cerevisiae*, which was then cultured on the appropriate non-inducing medium as previously described.^18^ Isolated colonies were suspended in water, diluted 10-fold, and then plated on inducing and non-inducing medium. Plates were imaged ~3 days later.

**Yeast Two-Hybrid and Co-Immunoprecipitation Assays.** The yeast two-hybrid system was carried out using Clontech Matchmaker Gold Yeast Two-Hybrid System (TaKaRa, Shiga, Japan). pGADT7 and pGBKT7 carrying various oyster gene fragments were transformed into the Y187 and Gold yeast strains, respectively. Y187 cells and Gold cells were cultured onto selective plates with synthetically defined medium (SD) lacking leucine (SD/-Leu) or tryptophan (SD/-Trp) separately. Three days later, positive yeast strains on SD/-Leu and SD/-Trp were collected and hybridized in 2X yeast extract peptone dextrose (YPDA) medium and selected on double drop-out SD/-Leu/-Trp medium. The blue hybridized clones growing on quadruple drop-out SD/-Ade/-His/-Leu/-Trp medium supplemented with X-α-Gal and Aureobasidin A (TaKaRa, Shiga, Japan) indicated potential protein interactions. For co-IP assays, HEK293T cells transfected with plasmids expressing the FLAG and Myc tags were harvested at 30 h post-transfection with cell lysis buffer (Beyotime, Jiangsu, China). Input samples were prepared from the cell lysate. The remaining lysate was mixed with anti-FLAG M2 magnetic beads (Sigma-Aldrich, St. Louis, MO, USA) and shaken gently on a roller shaker for 1-2 h. Subsequently, the magnetic beads were washed three times with cell lysis buffer and incubated with 2X SDS-PAGE loading buffer (TaKaRa, Shiga, Japan) for 8 min at 100 °C to elute the bound protein. Then, the beads were removed and immunoprecipitated proteins were analyzed using western blotting.

**Extraction of mitochondrial and cytoplasmic proteins and western blot analysis.** Mammalian and oyster mitochondria were isolated using a Cell Mitochondria Isolation Kit (Beyotime, Jiangsu, China) following the manufacturer’s protocol. The mitochondrial or cytoplasmic proteins were extracted from the prepared fractions with lysis buffer. For western blotting, proteins were resolved by 12% SDS-PAGE and transferred onto a 0.45 nm pore nitrocellulose membrane with the use of Semi-dry blotter (GenScript, Jiangsu, China). The membrane was then blocked with TBST (20 mmol/L Tris-HCL, 150 mmol/L NaCl, and 0.05% Tween-20) containing 5% skimmed milk at room temperature for 1 h, and then incubated with the appropriate antibodies at 4 ºC overnight, washed three times with TBST, incubated with peroxidase-labeled secondary antibodies at room temperature for 1 h, and finally washed five times with TBST. Western Lightning Plus-ECL substrate (PerkinElmer, Waltham, MA, USA) was then added to membranes, which were exposed to X-OMAT AR X-ray film (Eastman Kodak, Rochester, NY, USA).

**Subcellular localization.** pEGFP-N1 carrying distinct gene fragments were transfected into HeLa cells. HeLa cells were rinsed once with PBS 24 h later and then stained with 2 mg/mL Hoechst 33342 (Invitrogen, Carlsbad, CA, USA) for 10 min at 37 ºC. Subsequently, the cells were washed twice with PBS, stained with Alexa Fluor 594 (Life Technologies, Carlsbad, CA, USA) for 15 min at 37 ºC, washed three times with PBS, and then cultured in modified RPMI-1640 medium without fetal bovine serum. The visualization of protein subcellular localization was performed by confocal microscopy (Carl Zeiss, Oberkochen, Germany).

**Inhibitor treatments on oyster hemocytes.** Cell culture medium was replaced with fresh medium containing one of the inhibitors: PFT-α (100 μM), Z-LEHD-FMK (35 μM), or siRNA (30 μg/mL). Cells were incubated with PFT-α or Z-LEHD-FMK for 1 h, or siRNA for 24 h before UV light irradiation.

**Transcription activity assay.** HEK293T cells were used to examine Cg-p53 transcriptional activity. The region ~2 kb upstream of the *Cg-Bak*, *Cg-Bax*, *Cg-Bcl-2* and *Cg-Bcl-xl* start codons were considered as potential promoter regions and cloned to the pGL3-basic luciferase reporter plasmid to generate the *Cg-Bak*-Luc, *Cg-Bax*-Luc, *Cg-Bcl-2*-Luc and *Cg-Bcl-xl*-Luc plasmids. Cells were co-transfected with varying amounts of pRL-TK, luciferase reporter, pCMV-N-Myc-Cg-p53, and pCMV-N-Myc plasmids and luciferase activity was measured using Dual-Luciferase Reporter Assay System (Promega, Madison, MI, USA) 24 h after transfection.

**Assessment of caspase activity and apoptosis.** For caspase-3 and caspase-9 activity assays, HEK293T cells or oyster hemocytes were collected, lysed, and the concentration of total protein measured by Bradford method. Caspase activity was then monitored with the Caspase-3 Activity Assay Kit (Beyotime, Jiangsu, China) and Caspase-9 Activity Assay Kit (Beyotime, Jiangsu, China). Annexin V/PI immunostaining was used to assess apoptosis in cells using the FITC-Annexin V Apoptosis Detection Kit (BD Biosciences, San Jose, CA, USA) according to the manufacturer’s instructions. In brief, cells were washed with PBS, suspended with binding buffer, stained with FITC-Annexin V and PI, and analyzed with a FACS Calibur flow cytometer (BD, San Jose, CA, USA).

**JC-1 assay for mitochondrial membrane potential.** The mitochondrial membrane potential of oyster hemocytes was evaluated with the JC-1 Mitochondrial Membrane Potential Detection Kit (Beyotime, Jiangsu, China) according to the manufacturer’s protocol. Briefly, hemocytes that mixed with 10 μM CCCP for 20 min before JC-1 staining served as a positive control. Hemocytes in each well were collected and resuspended with 1 mL modified L15 medium and then mixed with JC-1 staining solution. Twenty minutes later, the cells were rinsed twice with 1X staining buffer and analyzed with a Varioskan Flash multimode reader (Thermo Fisher Scientific, Sunnyvale, CA, USA).

**Statistical analysis**. Data were analyzed by Student’s t-test or one-way ANOVA to determine the differences among different groups. *P* < 0.05 was considered statistically significant.

**Acknowledgements**

This study was supported by the National Basic Research Program of China (973 Program, No.2010CB126402), the National Natural Science Foundation of China (31302219), the National High Technology Research and Development Program (863 program, 2012AA10A405), the Earmarked Fund for Modern Agro-industry Technology Research System (CARS-48), and Taishan Scholars Climbing Program of Shandong.

**Conflict of Interest**

There is no conflict of interest in this work.

**References**

1. Guofan Z, Xiaodong F, Ximing G, Li L, Ruibang L, Fei X *et al*. The oyster genome reveals stress adaptation and complexity of shell formation*.* *Nature* 2012; **490**: 49-54.

2. Francis MH, Brent F, Snimar G, Inna MS. Apoptosis as a host defense mechanism in *Crassostrea virginica* and its modulation by *Perkinsus marinus.* *Fish Shellfish Immunol* 2010; **29**: 247–257.

3. Sokolova IM, Evans S, Hughes FM. Cadmium-induced apoptosis in oyster hemocytes involves disturbance of cellular energy balance but no mitochondrial permeability transition*.* *J Exp Biol* 2004; **207**: 3369-3380.

4. Foster B, Grewal S, Graves O, Hughes FM, Sokolova IM. Copper exposure affects hemocyte apoptosis and *Perkinsus marinus* infection in eastern oysters *Crassostrea virginica* (Gmelin)*.* *Fish Shellfish Immunol* 2011; **31**: 341–349.

5. Renault T, Faury N, Barbosa-Solomieu V, Moreau K. Suppression substractive hybridisation (SSH) and real time PCR reveal differential gene expression in the Pacific cupped oyster, *Crassostrea gigas*, challenged with *Ostreid herpesvirus* 1*.* *Dev Comp Immunol* 2011; **35**: 725-735.

6. Zhang L, Hou R, Su H, Hu X, Wang S, Bao Z. Network analysis of oyster transcriptome revealed a cascade of cellular responses during recovery after heat shock*.* *Plos One* 2012; **7**: e35484.

7. Zhao X, Yu H, Kong L, Li Q. Transcriptomic responses to salinity stress in the Pacific Oyster *Crassostrea gigas.* *Plos One* 2012; **7**: e46244-e46244.

8. Goldstein JC, Waterhouse NJ, Juin P, Evan GI, Green DR. The coordinate release of cytochrome *c* during apoptosis is rapid, complete and kinetically invariant*.* *Nat Cell Biol* 2000; **2**: 156-162.

9. Peng L, Deepak N, Imawati B, Srinivasa MS, Manzoor A, Emad SA *et al*. Cytochrome c and dATP-dependent formation of Apaf-1/caspase-9 complex initiates an apoptotic protease cascade*.* *Cell* 1997; **91**: 479–489.

10. Kluck RM, Bossy-Wetzel E, Green DR, Newmeyer DD. The release of cytochrome c from mitochondria: a primary site for Bcl-2 regulation of apoptosis*.* *Science* 1997; **275**: 1132-1136.

11. Kluck RM, Seamus JM, Hoffman BM, Zhou JS, Green DR, Newmeyer DD. Cytochrome c activation of CPP32-like proteolysis plays a critical role in a *Xenopus* cell-free apoptosis system*.* *Embo Journal* 1997; **16**: 4639-4649.

12. Robertson AJ, Croce J, Carbonneau S, Voronina E, Miranda E, Mcclay DR *et al*. The genomic underpinnings of apoptosis in *Strongylocentrotus purpuratus.* *Dev Biol* 2006; **300**: 321–334.

13. Robb SMC, Eric R, Alejandro SA. SmedGD: the *Schmidtea mediterranea* genome database*.* *Nucleic Acids Res* 2008; **36**: D599–D606.

14. Sullivan JC, Ryan JF, Watson JA, Webb J, Mullikin JC, Rokhsar D *et al*. StellaBase: the *Nematostella vectensis* genomics database*.* *Nucleic Acids Res* 2006; **volume 34**: D495-499.

15. Wiens M, Krasko A, Müller CI, Müller WE. Molecular evolution of apoptotic pathways: cloning of key domains from sponges (Bcl-2 homology domains and death domains) and their phylogenetic relationships*.* *J Mol Evol* 2000; **50**: 520-531.

16. Hu Y, Ding L, Spencer DM, Núñez G. WD-40 repeat region regulates Apaf-1 self-association and procaspase-9 activation*.* *J Biol Chem* 1998; **273**: 33489-33494.

17. Rodriguez A, Oliver H, Zou H, Chen P, Wang X, Abrams JM. Dark is a Drosophila homologue of Apaf-1/CED-4 and functions in an evolutionarily conserved death pathway*.* *Nat Cell Biol* 1999; **1**: 272-9.

18. Bender CE, Patrick F, Tait SWG, Fabien L, Mcstay GP, Tupper DO *et al*. Mitochondrial pathway of apoptosis is ancestral in metazoans*.* *Proc Natl Acad Sci USA* 2012; **109**: 4904-9.

19. Lee EF, Clarke OB, Marco E, Zhiping F, Speed TP, Tchoubrieva EB *et al*. Discovery and molecular characterization of a Bcl-2-regulated cell death pathway in schistosomes*.* *Proc Natl Acad Sci USA* 2011; **108**: 6999-7003.

20. Oberst A, Bender C, Green DR. Living with death: the evolution of the mitochondrial pathway of apoptosis in animals*.* *Cell Death Differ* 2008; **15**: 1139-1146.

21. Zhang L, Li L, Zhang G. Gene discovery, comparative analysis and expression profile reveal the complexity of the *Crassostrea gigas* apoptosis system*.* *Dev Comp Immunol* 2011; **35**: 603–610.

22. Tao Q, Baoyu H, Linlin Z, Li L, Fei X, Wen H *et al*. Identification and functional characterization of two executioner caspases in *Crassostrea gigas.* *Plos One* 2014; **9**: e89040.

23. Tao Q, Zhang L, Wei W, Huang B, Li Y, Zhu Q *et al*. Characterization of an inhibitor of apoptosis protein in *Crassostrea gigas* clarifies its role in apoptosis and immune defense*.* *Dev Comp Immunol* 2015; **51**: 74–78.

24. Alejandro R, Noelia EC, Sonia D, Antonio F, Beatriz N. New insights into the apoptotic process in mollusks: characterization of caspase genes in *Mytilus galloprovincialis.* *Plos One* 2011; **6**: e17003-e17003.

25. Estévez-Calvar N, Romero A, Figueras A, Novoa B. Genes of the mitochondrial apoptotic pathway in *Mytilus galloprovincialis.* *Plos One* 2013; **8**: e61502-e61502.

26. Margherita L, Barbara P, Nikola S, Mihai C, Beate S, Tina K *et al*. The molecular cell death machinery in the simple cnidarian *Hydra* includes an expanded caspase family and pro-and anti-apoptotic Bcl-2 proteins*.* *Cell Res* 2010; **20**: 812-825.

27. Hsieh SY, Hsu CY, He JR, Liu CL, Lo SJ, Chen YC *et al*. Identifying apoptosis-evasion proteins/pathways in Human hepatoma cells via induction of cellular hormesis by UV irradiation*.* *J Proteome Res* 2009; **8**: 3977-86.

28. Zhang Z, Liu L, Wu S, Xing D. Drp1, Mff, Fis1, and MiD51 are coordinated to mediate mitochondrial fission during UV irradiation-induced apoptosis*.* *FASEB J* 2016; **30**: 466-476.

29. Gervais O, Renault T, Arzul I. Induction of apoptosis by UV in the flat oyster, *Ostrea edulis.* *Fish Shellfish Immunol* 2015; **46**: 232-42.

30. Von AO, Waterhouse NJ, Kuwana T, Newmeyer DD, Green DR. The 'harmless' release of cytochrome c*.* *Cell Death Differ* 2000; **7**: 1192-1199.

31. Jürgensmeier JM, Krajewski S, Armstrong RC, Wilson GM, Oltersdorf T, Fritz LC *et al*. Bax- and Bak-induced cell death in the fission yeast *Schizosaccharomyces pombe.* *Mol Biol Cell* 1997; **8**: 325–339.

32. Sattler M, Liang H, Nettesheim D, Meadows RP, Harlan JE, Eberstadt M *et al*. Structure of Bcl-xL-Bak peptide complex: recognition between regulators of apoptosis*.* *Science* 1997; **275**: 983-986.

33. Rebeca M, Pablo B, Josep VP, Berta F, Sergi B, Beatriz N *et al*. Transcriptomics of In Vitro Immune-Stimulated Hemocytes from the Manila Clam *Ruditapes philippinarum* Using High-Throughput Sequencing*.* *Plos One* 2012; **7**: 410-410.

34. Philipp EE, Kraemer L, Melzner F, Poustka AJ, Thieme S, Findeisen U *et al*. Massively parallel RNA sequencing identifies a complex immune gene repertoire in the lophotrochozoan *Mytilus edulis.* *Plos One* 2012; **7**: e33091.

35. Boris Z, Guido K. Apoptosis and genomic instability*.* *Nat Rev Mol Cell Biol* 2004; **5**: 752-762.

36. Varkey J, Chen P, Jemmerson R, Abrams JM. Altered cytochrome c display precedes apoptotic cell death in *Drosophila.* *J Cell Biol* 1999; **144**: 701-10.

37. Dorstyn L, Read S, Cakouros D, Huh JR, Hay BA, Kumar *et al*. The role of cytochrome c in caspase activation in *Drosophila melanogaster* cells*.* *J Cell Biol* 2002; **156**: 1089-98.

38. Zimmermann KC, Ricci JE, Droin NM, Green DR. The role of ARK in stress-induced apoptosis in *Drosophila* cells*.* *J Cell Biol* 2002; **156**: 1077-87.

39. Sato M, Matsushima K, Kawanami H, Ikuhsima Y. The two cytochrome c species, DC3 and DC4, are not required for caspase activation and apoptosis in *Drosophila* cells*.* *J Cell Biol* 2004; **167**: 405-10.

40. Ranger AM, Malynn BA, Korsmeyer SJ. Mouse models of cell death*.* *Nat Genet* 2001; **28**: 113-118.

41. Hu Y, Ding L, Spencer DM, Núñez G. WD-40 repeat region regulates Apaf-1 self-association and procaspase-9 activation*.* *J Biol Chem* 1998; **273**: 33489-94.

42. Hsu YT, Wolter KG, Youle RJ. Cytosol-to-membrane redistribution of Bax and Bcl-XL during apoptosis*.* *Proc Natl Acad Sci USA* 1997; **94**: 3668-3672.

43. Wolter KG, Hsu YT, Smith CL, Nechushtan A, Xi XG, Youle RJ. Movement of Bax from the cytosol to mitochondria during apoptosis*.* *J Cell Biol* 1997; **139**: 1281–1292.

44. Dhyan C, Grace C, Daniel PT, Tang DG. Bax-dependent regulation of Bak by voltage-dependent anion channel 2*.* *J Biol Chem* 2005; **280**: 19051-19061.

45. Li Y, Zhang L, Qu T, Li L, Zhang G. Characterization of oyster voltage-dependent anion channel 2 (VDAC2) suggests its involvement in apoptosis and host defense*.* *Plos One* 2016; **11**: e0146049.

46. Chipuk JE, Moldoveanu T, Llambi F, Parsons MJ, Green DR. The BCL-2 family reunion*.* *Mol Cell* 2010; **37**: 299-310.

47. Bonsu K, Chengyu L, Jung JU, Byung-Ha O. Evidence that inhibition of BAX activation by BCL-2 involves its tight and preferential interaction with the BH3 domain of BAX*.* *Cell Res* 2011; **21**: 627-641.

48. Speidel D. Transcription-independent p53 apoptosis: an alternative route to death*.* *Trends Cell Biol* 2009; **20**: 14-24.

49. Zhang L, Li L, Guo X, Litman GW, Dishaw LJ, Zhang G. Massive expansion and functional divergence of innate immune genes in a protostome*.* *Sci Rep* 2015; **5**: 8693.

50. Li C, Tao Q, Huang B, Peng J, Wen H, Que H *et al*. Cloning and characterization of a novel caspase-8 -like gene in *Crassostrea gigas.* *Fish Shellfish Immunol* 2015; **46**: 486-492.

51. Leist M, Marja J. Four deaths and a funeral: from caspases to alternative mechanisms*.* *Nat Rev Mol Cell Biol* 2001; **2**: 589-598.

52. Feng Y, Yang Z, Ziniu Y. Characteristics and expression patterns of the lipopolysaccharide-induced TNF-α factor (LITAF) gene family in the Pacific oyster, *Crassostrea gigas.* *Fish Shellfish Immunol* 2012; **33**: 899–908.

53. Du Y, Zhang L, Huang B, Guan X, Li L, Zhang G. Molecular cloning, characterization, and expression of two myeloid differentiation factor 88 (Myd88) in Pacific Oyster, *Crassostrea gigas.* *J World Aquacult Soc* 2013; **44**: 759-774.

**Figure Legends**

**Fig. 1 | Analysis of apoptosis, mitochondrial membrane potential (MMP), and cytochrome *c* release in UV-irradiated hemocytes of *C. gigas*.** (A) Oyster hemocytes were irradiated with UV light and apoptosis was monitored by Annexin V/propidium iodide (PI) immunostaining at 20 h post-irradiation (hpi). Data are shown as the mean ± SD (N = 6). ***P* < 0.01. (B) Oyster hemocytes were UV-irradiated or treated with 10 μM carbonylcyanide-p-chlorophenyl hydrazone (CCCP), and hemocyte MMP was measured by JC-1 assay. Irradiated cells were sampled and measured at 6 hpi, 9 hpi, and 24 hpi. CCCP-treated and untreated/non-irradiated cells served as positive and negative controls, respectively. Lower 590/530 nm ratios refer to lower ΔΨm. N = 6 per time point (including negative control and CCCP group), data are shown as the mean ± SD. (C) Cytosolic proteins were extracted from irradiated (24 hpi) and non-irradiated oyster hemocytes and analyzed by western blot using anti-cytochrome *c* and anti-actin antibodies.

**Fig. 2 | Determination of caspase 9 and caspase 3 involvement in mitochondrial apoptosis in *C. gigas*.** (A) Caspase 9 and (B) Caspase 3 activity in oyster hemocytes was measured at 20 hpi. Non-irradiated cells were used as a negative control. Data are displayed as the mean ± SD (N = 6). ***P* < 0.01. (C) Cytosolic extracts from oyster hemocytes were treated with cytochrome *c* (cyt *c*), Z-LEHD-FMK and dATP. Caspase 9 activity was measured at 2 h post cyt *c* treatment (10 μM of horse cyt *c* or oyster cyt *c*). In Cg-cyt *c* + Z-LEHD-FMK group, the cytosolic extracts were preincubated with Z-LEHD-FMK (35 μM) for 1 h and then treated with Cg-cyt *c*. For Cg-cyt *c* + dATP treatment, the extracts were incubated with both Cg-cyt *c* and dATP (1 mM) for 2 h before activity assessment. Different letters indicate significant differences at *P* ˂ 0.05, whereas conditions annotated with the same letter were not significantly different. (D) Caspase 3 activity in oyster cytosolic extracts was monitored at different time points after treatment with 10 μM oyster cyt *c* with or without 1 mM dATP. Untreated extract served as a negative control. (E) Caspase 3 activity was examined in irradiated and non-irradiated hemocytes pretreated with Z-LEHD-FMK, DMSO vehicle, or left untreated at 20 hpi. Different small letters denote significant differences at *P* < 0.05 while same letters denote not. All of the results in Figure 2 are shown as the mean ± SD (N = 6).

**Fig. 3** **| Sequence analysis of oyster Bcl-2 family proteins.** (A) Small motif architecture of Bcl-2 family proteins in *Crassostrea gigas*. *C. gigas* Bcl-2 family proteins contained at least three of four conserved sequence motifs known as Bcl-2 homology domains (BH1–BH4, indicated in the figure). Cg-Bak and Cg-Bax also contained the transmembrane domain (gray box). (B) Neighbor-joining phylogenetic tree of Bcl-2 family homologs from different vertebrate and invertebrate species. The neighbor-joining tree constructed by the MEGA program was based on the sequences of four Bcl-2 family proteins in *C. gigas*, along with Bcl-2 family homologs from other species, including Bcl-2 homologs from *Homo sapiens* (NP_000624.2), *Xenopus laevis* (NP_001139565.1), *Danio rerio* (NP_001025424.1 and NP_571882.1), *Drosophila melanogaster* (NP_523702.1), *Caenorhabditis elegans* (NP_499284.1), *Lottia gigantea* (XP_009061217.1 and XP_009067239.1), *Schistosoma japonicum* (CAX69465.1), and *Hydra vulgaris* (NP_001274311.1); Bcl-xL homologs from *Homo sapiens* (CAA80661.1), *Xenopus laevis* (NP_001082147.1), and *Danio rerio* (NP_571882.1), Bak homologs from *Homo sapiens* (NP_001179.1), *Xenopus laevis* (NP_001089587.1), *Lottia gigantea* (XP_009064284.1), *Schistosoma japonicum* (CAX70134.1), *Schistosoma mansoni* (CCD80772.1), *Schmidtea mediterranea* (AEX93474.1, AEX93475.1 and AEX93476.1), and *Hydra vulgaris* (NP_001296708.1), and Bax homologs from *Xenopus laevis* (NP_001079104.1), *Danio rerio* (NP_571637.1), and *Schistosoma mansoni* (CCD81694.1).

**Fig. 4 | Functional analysis of oyster Bcl-2 family proteins.** (A) Plasmids expressing the indicated oyster or human Bcl-2 family proteins were transformed into *Saccharomyces cerevisiae* to determine their effect on yeast cell growth. Yeast were plated in 10-fold serial dilutions and induced to express the transformed genes. (B and C) HEK293T cells transfected with various plasmids (indicated in the figure) were treated with 146 μM H_2_O_2_ for 40 min. Caspase 3 activity was examined 20 hpi by spectrophotometric detection of the chromophore p-nitroaniline (pNA) after cleavage from the labeled substrate DEVD-pNA. Data are shown as the mean ± SD (N = 3). Different small letters indicate significant differences (*P* < 0.05) while the same letter indicated not. (D) Expression patterns of oyster Bcl-2 family genes following irradiation were detected. Data are displayed as the mean ± SD (N = 3). **P* ˂ 0.05, ** *P* ˂ 0.01. (E and F) Analysis of *Cg-Bak* and *Cg-Bax* mRNA levels in oyster hemocytes after siRNA transfection was used to confirm the RNAi knockdown. PBS-treated hemocytes were used as a negative control. Hemocytes in the negative RNAi group were treated with siRNA sequences as indicated in Method section. Data are displayed as the mean ± SD (N = 3). Different small letters denote significant differences (*P* < 0.05) and the same letter denote not. (G and H) SiRNA *Cg-Bak-*treated and *Cg-Bax*-treated hemocytes were UV-irradiated and apoptosis levels were measured at 3 hpi and 24 hpi. Data are shown as the mean ± SD (N = 6). Different small letters referred to differences at *P* ˂ 0.05.

**Fig. 5 | Investigation on the apoptotic regulatory functions of Cg-Bak and Cg-Bax.** (A) HeLa cells were transfected with Cg-Bak-EGFP or pEGFP-N1 empty vector and UV-irradiated or left untreated. Cell nuclei were stained with Hoechst 33342 (blue) and Alexa Fluor 633 (red) or MitoView 633 (red) to stain the cell membrane or mitochondria, respectively, as indicated in the figure. The green fluorescent signal indicated the distribution of overexpressed proteins. (B) HeLa cells were transfected with Cg-Bax-EGFP or pEGFP-N1 empty vector (green) and then UV-irradiated or left untreated. Cell nuclei were stained with Hoechst 33342 (blue). (C) Cg-Bak-EGFP and Cg-Bax-EGFP were overexpressed in HEK293T cells, which were then subjected to UV irradiation. Cytosolic and mitochondrial extracts from non-irradiated and irradiated cells were examined by western blot with anti-GFP antibody. (D) Isolated mitochondria from oyster cells were incubated with storage buffer, CaCl_2_ (300 μM), recombinant Cg-Bak protein (8 μM), or recombinant Cg-Bax (8 μM) at room temperature for 30 min prior to centrifugation. The distributions of cytochrome *c* in the supernatant (S) and precipitate (P) were then analyzed by western blot.

**Fig. 6 | Interactions of oyster Bcl-2 anti-apoptotic and pro-apoptotic subfamily proteins.** (A) Hybridized yeast containing both tested proteins was cultured on Quadruple drop-out (QDO) plates and imaged 3-5 days later. Blue colonies indicate a potential direct interaction. (B) Names and structures of the truncated mutant proteins used in this study. The BH1 (diagonal), BH2 (dots), BH3 (solid black), BH4 (grid), and transmembrane (solid gray) domains are shown. Numbers denote the nucleic acid location. (C) Interaction between distinct truncated mutant proteins and oyster Bcl-2 family proteins detected by yeast two-hybrid system. (D) Tested proteins carrying either FLAG- or Myc-tags were co-overexpressed in HEK293T cells and interactions determined by co-immunoprecipitation assays using M2 anti-FLAG antibody. (Top) anti-Myc pulldown, (Middle) western blot using anti-Myc antibody, and (Bottom) western blot using anti-FLAG antibody. (E) Interaction between distinct truncated mutant proteins and oyster Bcl-2 family proteins detected by co-IP assays.

**Fig. 7 | Involvement of Cg-p53 in mitochondrial apoptosis in *C. gigas*.** (A) Expression pattern of *Cg-p53* in oyster hemocytes upon UV light irradiation. (B) Effect of PFT-α treatment on *Cg-p53* expression. Oyster hemocytes were incubated with 100 μM PFT-α for 1 h and *Cg-p53* expression was analyzed 3 h and 6 h later. The data in (A) and (B) are shown as the mean ± SD (N = 3). (C) Apoptosis was monitored by annexin V/PI immunostaining in hemocytes following treatment as indicated. Data are displayed as the mean ± SD (N = 6). **P* ˂ 0.05, ***P* ˂ 0.01.

**Fig. 8 | Role of Cg-p53 on regulating Bcl-2 family activities in mitochondrial apoptosis pathway in *C. gigas*.** (A) Schematic diagram showing the regions of *Cg-Bak*, *Cg-Bax*, *Cg-Bcl-2*, and *Cg-Bcl-xl* considered as presumed promoter regions (gray) and subcloned into the pGL3-basic plasmid to construct the luciferase reporter. (B) Transactivation activity of Cg-p53 to *Cg-Bak*. The luciferase reporter containing presumed promoter region of *Cg-Bak*, pCMV-Cg-p53, and pRL-TK (as an internal control) were co-transfected into HEK293T cells and luciferase activity was measured 24 h later. Transactivation activity is expressed as fold increase over the control group (far left column) with no pCMV-Cg-p53. (C) Transactivation activity of Cg-p53 to *Cg-Bax*. (D) Transactivation activity of Cg-p53 to *Cg-Bcl-2*. (E) Transactivation activity of Cg-p53 to *Cg-Bcl-xl*. (F) *Cg-Bak* and (G) *Cg-Bax* expression in PFT-α treated hemocytes following irradiation. Data in (B), (C), (D), (E), (F) and (G) are shown as the mean ± SD (N = 3). **P* ˂ 0.05, ***P* ˂ 0.01. (H) Interactions between Cg-p53 and the four oyster Bcl-2 family proteins determined by co-IP assays in HEK293T cells. (Top) The IP samples against anti-Myc antibody. (Middle) The input samples against anti-Myc antibody. (Bottom) The input samples against anti-FLAG antibody.

**Supporting Information**

**S1 Fig.** **Transmission electron microscopy analysis of morphological modifications in oyster hemocytes induced by UV irradiation.** (A) Control cell without morphological modification. (B-D) Apoptotic cells.

**S2 Fig. Tissue distributions Bcl-2 family gene transcripts in *C. gigas*.**

**S3 Fig. Expression pattern of oyster Bcl-2 family gene transcripts in ostreid herpesvirus 1 (OsHV-1)-infected hemolymph.**

**S4 Fig. Expression pattern of oyster Bcl-2 family gene transcripts in *Vibrio alginolyticus*-infected hemolymph.**

**S5 Fig. Expression pattern of oyster Bcl-2 family gene transcripts in hemolymph upon air exposure.**

**S6 Fig.** **Expression pattern of oyster Bcl-2 family gene transcripts in hemolymph after heat shock treatment (35 ºC) for 1 h.**

**S7 Fig. Expression patterns of *Cg-Bcl-2* and *Cg-Bcl-xl* upon UV irradiation after PFT-α treatment in oyster hemocytes.**

**S8 Fig. Percentage of dead cells (PI-positive and annexin V-FITC-negative) among oyster hemocytes subjected to UV irradiation and PFT-α treatment.**
